# Short Photoluminescence Lifetimes Linked to Crystallite Dimensions, Connectivity, and Perovskite Crystal Phases

**DOI:** 10.1021/acs.jpcc.1c08867

**Published:** 2022-02-15

**Authors:** Raquel Chuliá-Jordán, Emilio J. Juarez-Perez

**Affiliations:** † Instituto de Ciencia de los Materiales, Universitat de València, C/Catedrático J. Beltrán, 2, Paterna 46980, Spain; ‡ ARAID Foundation, Instituto de Nanociencia y Materiales de Aragón (INMA), CSIC - Universidad de Zaragoza, Zaragoza 50009, Spain

## Abstract

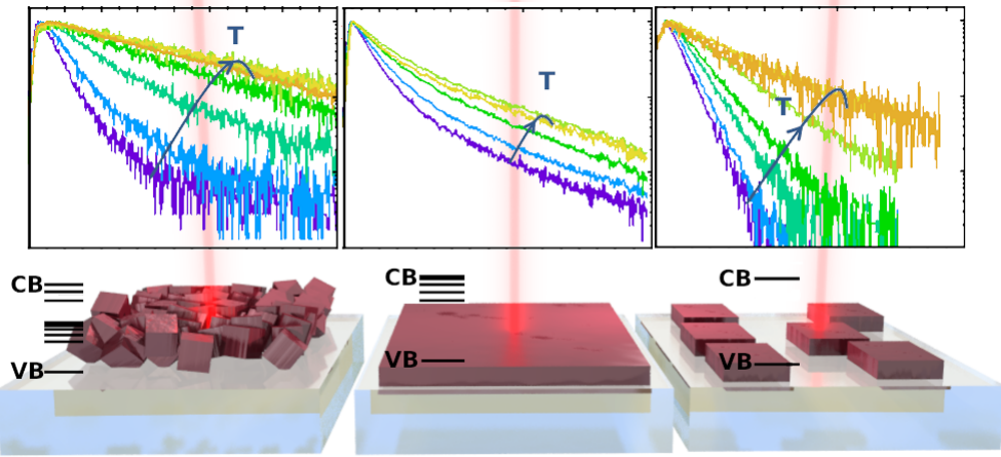

Time-correlated single photon counting has been conducted to gain further insights into the short photoluminescence lifetimes (nanosecond) of lead iodide perovskite (MAPbI_3_) thin films (∼100 nm). We analyze three different morphologies, compact layer, isolated island, and connected large grain films, from 14 to 300 K using a laser excitation power of 370 nJ/cm^2^. Lifetime fittings from the Generalized Berberan-Santos decay model range from 0.5 to 6.5 ns, pointing to quasi-direct bandgap emission despite the three different sample strains. The high energy band emission for the isolated-island morphology shows fast recombination rate centers up to 4.8 ns^–1^, compared to the less than 2 ns^–1^ for the other two morphologies, similar to that expected in a good quality single crystal of MAPbI_3_. Low-temperature measurements on samples reflect a huge oscillator strength in this material where the free exciton recombination dominates, explaining the fast lifetimes, the low thermal excitation, and the thermal escape obtained.

## 1 Introduction

Over the past years, a large number of studies have shown the technological versatility of lead halide perovskites due to their remarkable physical properties.^1−4^ These compounds have allowed obtaining an unprecedented progress in light-to-energy conversion efficiencies^5−7^ and efficient materials for light-emitting diodes (LEDs).^8^

Despite the huge number of implementations in a multitude of applications, there is a lack of understanding of the origin of the remarkable properties of perovskites.^9−11^ For example, it is known that the photon emission process (photoluminescence, PL) is more efficient in a direct bandgap semiconductor than in an indirect bandgap semiconductor (in this case, a phonon is required to emit a photon).^12,13^ Hence, the long carrier lifetime (τ) measured in perovskites is not complete understood if we assume them to be direct bandgap semiconductors with efficient absorption.^14−20^ That is to say, there is a contradictory behavior in the properties that the perovskites exhibit if we consider that the absorption and emission of photons are ruled by the same transition matrix element.

Since long lifetimes often indicate materials with long diffusion lengths (), one of the main intensively explored topics is the preservation of carrier diffusion length in perovskite materials.^21−26^ It has been shown that the lead iodide perovskite film has a diffusion length about two times longer than that of the lead bromide perovskite film.^21^ Even between the same perovskite material, the diffusion length depends on the sample preparation method,^21^ as each deposition/growth method comes with some characteristic defects in a microscale. As it might influence the results, these characteristic defects, due to the preparation method, are typically determined using static/lifetime photoluminescence mapping,^27^ or at least by proving the reproducibility of results on multiple samples. For routing testing of the carrier diffusion length, a laser grating technique has been proposed, instead of joint measurements of mobility and photoluminescence lifetime.^21^

Taking into account that the diffusion coefficient (*D*) is an intrinsic property of a crystal, the most common standard model among the scientific community to determine *D* is to assume perfect extraction or Dirichlet contour conditions *n* = 0 at the position *x*, where the selective contact is located.^28^ Recently, it was reported that a model with three slabs of different thicknesses of the same material (the same *D*) suffer different direct electron population detection decays.^29^ The different recombination kinetics due to their different thicknesses (concentrations of carriers) showed that thinner slabs have shorter lifetimes than thicker areas, and their dependence on thickness is remarkable.

In order to get further insights into short photoluminescence lifetimes, we have investigated the dynamics of the recombination pathways of the same material (therefore having the same *D*) in three different morphologies of lead iodide perovskites films at different temperatures ranging from 14 to 300 K and at a laser excitation power of 370 nJ/cm^2^. With the aim of avoiding an arbitrary number of exponential functions to describe the time-resolved PL (TRPL) spectra decay, the Generalized Berberan-Santos decay equation (GBSe) was used.^30^ The distribution of recombination rate constants (*k*) depending on the probability density function (*H*) has allowed obtaining both qualitative and quantitative information, such as the fast recombination centers and the FWHM, which has shown that at the high energy band emissions for the isolated-island configuration, the fast recombination rate centers are up to 4.8 ns^–1^, which explain the fast lifetimes, the low thermal excitation, and the thermal escape obtained. Our observations corroborate that the isolated-island configuration behaves as a semiconductor with low density of impurities that inhibit bound exciton recombination. In fact, its behavior at low temperatures would be comparable to that expected in a good quality single crystal of MAPbI3, and the PL kinetics measured is clearly associated with the “O”-phase free exciton recombination.

## 2 Methods and Materials

### 2.1 Sample Preparation and Description

The three different morphologies of CH_3_NH_3_PbI_3_ lead iodide perovskites (hereafter MAPbI_3_) films are as follows: (i) 550 nm-thick continuous multilayers of small grains (hereafter 550 nm-CS); (ii) 352 nm-thick continuous one layer of large grains (hereafter 352 nm-CL); and (iii) low density of isolated 103–200 nm-thin islands of small grains (hereafter 103 nm-IS).

**Figure 1:**
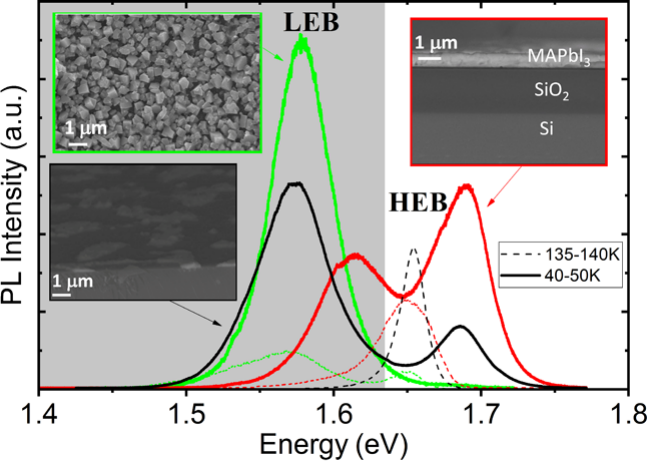
Morphological characterizations by SEM of the three selected morphologies and their corresponding PL spectra to show the low energy band (LEB) and the high energy band (HEB) at two different temperatures (40–50 K dashed line and 135–140 K solid line) and at a laser excitation power of 370 nJ/cm^2^. (Green) one layer of large grains (352 nm-CL), (red) multilayers of small grains (550 nm-CS), and (black) one layer of islands of small grains (103 nm-IS).

The size of the small grain of 550 nm-CS and 103 nm-IS is 103 ± 9 nm, while the size of the large grain of 352 nm-CL is 352 ±38 nm (see Figure 1). The length of each isolated island is approximately 1 μm (1000 nm ±120 nm), and although most of the isolated islands are only formed by a monolayer of small grains, a second layer of small grains could not be ruled out.

Samples have been grown using a spin-coating solution processing method. The details of the process can be found in refs (1) and (4):

#### 2.1.1 352 nm-CL

An approximately 40 nm-thick layer of TiO_2_ was deposited on the substrate fluorine-doped tin oxide-coated glass. A 200 nm TiO_2_ mesoporous layer was placed on this TiO_2_ buffer layer. MAPbI_3_ “cuboid-like” films of 352 nm thickness were deposited inside a glovebox by spin-coating 30 μL of a 1.082 M solution of PbI_2_ in dimethylformamide at 500 rpm for 5 s. The PbI_2_ film was then dipped for 1 min in a CH_3_NH_3_I solution in isopropanol (0.044 M).

#### 2.1.2 550 nm-CS and 103 nm-IS

The structure of the samples consists of a substrate of SiO_2_ (2 μm), a TiO_2_ layer (40 nm), an active layer of MAPbI_3_ (550 nm for 550 nm-CS and 100–200 nm for 103 nm-IS), and a poly-methyl methacrylate (PMMA) capping layer (1 μm) in order to protect the sample from oxidation. 103 nm-IS was achieved by diluting to 10% (w/w) the spin-coated perovskite solution of 40% used for the other two morphologies 550 nm-CS and 352 nm-CL.

### 2.2 Experimental Setup^1,4^

The cold finger of a commercial closed-cycle compressed helium cryostat (ARS DE-202) was used to characterize the samples. This cryo-generator has a heating resistance and a thermometer, with which the temperature can be controlled from 10 K to room temperature. For time-integrated PL measurements, we used a continuous wave laser diode at 405 nm. In time-resolved PL (TRPL) experiments, we used for excitation a 200 fs pulsed Ti:sapphire (Coherent Mira 900D, 76 MHz of repetition rate) laser doubled to 400 nm with a BBO crystal. The backscattered PL signal was dispersed by a double 0.3 m focal length grating spectrograph/spectrometer (1200 g/mm with 750 nm blaze) and detected by an Andor Newton 970 EMCCD camera (for time-integrated PL spectra) and by a Si Micro Photon Device (MPD) single photon avalanche diode (SPAD) photodetector connected through a multimode optical fiber to the monochromator (for time-resolved PL spectra); the SPAD was attached to a time correlated single photon counting electronic board (TCC900 from Edinburgh Instruments).

## 3 Results and Discussion

### 3.1 Time-Resolved Photoluminescence Measurements Depending on Temperature

**Figure 2:**
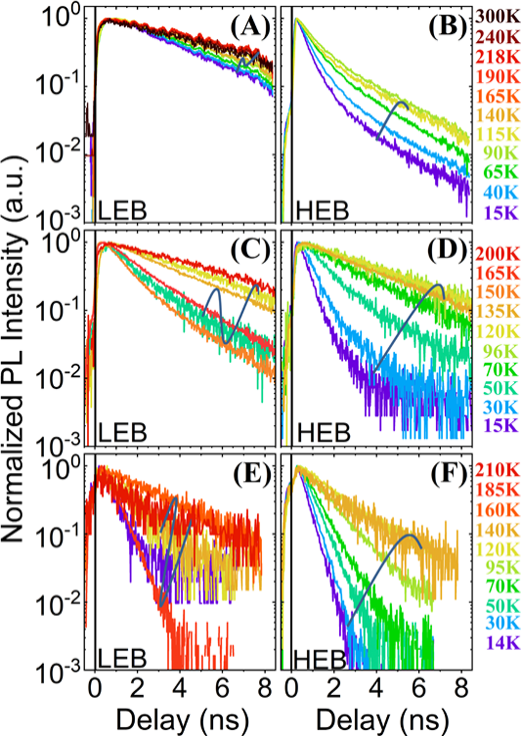
(a–f) Temperature dependence of the time-resolved photoluminescence (PL) emission measured for the three different morphologies: (a, b) one layer of large grains (352 nm-CL), (c, d) multilayers of small grains (550 nm-CS), and (e, f) one layer of islands of small grains (103 nm-IS) at 370 nJ/cm^2^. The blue curves represent the evolution of the emission by increasing the temperature, which is different in the HEB and LEB.

**Figure 3:**
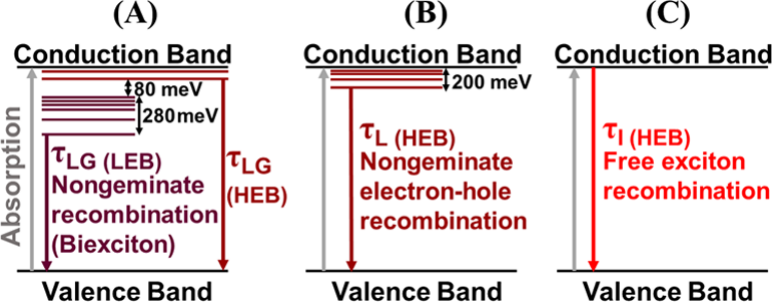
Low-temperature recombination channels in MAPbI_3_ for the three morphologies: (a) one layer of large grains (352 nm-CL), (b) multilayers of small grains (550 nm-CS), and (c) one layer of islands of small grains (103 nm-IS). At low temperatures, the grain size of the layer of large grains is responsible for the appearance of the dual emission (see Figure 1 and ref (4)). As the island morphology does not exhibit strain inhomogeneities related to the formation of different tetragonal-defective domains, a high excitation fluency of 370 nJ/cm^2^ was chosen to fill most of these defective tetragonal-phase sites of the continuous samples (550 nm-CS and 352 nm-CL). This ensures a similar temperature dependence on the lifetime for “O”-excitons for the three morphologies.

To give new insights into the connection between the morphology of the lead halide perovskites’ absorber layer/islands and their photophysical properties,^31^ we have measured the time-resolved photoluminescence (TRPL) for both the low-energy (LEB) and the high-energy (HEB) band emissions (see Figure 1). These bands have been associated to exciton recombination in tetragonal and orthorhombic phases, respectively.^32−34^ These bands coexist in a certain temperature range, the HEB dominating at low *T* and the LEB at high *T*, as defined by the phase transitions/discontinuities. Only for 103 nm-IS, there is no coexisting crystallographic phases. The details of the PL spectra can be found in refs (1) and (4). Figure 2a–f shows the representative groups of normalized PL transients registered under relatively high excitation fluencies (370 nJ/cm^2^) for the three types of morphologies studied: 550 nm-CS, 352 nm-CL, and 103 nm-IS. This high excitation fluency of 370 nJ/cm^2^ was chosen because most of the defective tetragonal-phase sites of the continuous samples (550 nm-CS and 352 nm-CL) are filled (see Figure 3). In this way, the continuous samples (550 nm-CS and 352 nm-CL) exhibit a similar temperature dependence on the lifetime for “O”-excitons to the island-like sample (103 nm-IS).^1^ The normalized TRPL of the LEB (HEB) band is presented in the left (right) column.

In general, it is observed that the time-resolved normalized PL intensity is strongly dependent on the different morphologies (550 nm-CS, 352 nm-CL, and 103 nm-IS). For example, the PL intensity of 103 nm-IS (see Figure 2e,f) exhibits shorter lifetimes than the other two configurations (see Figure 2a–d). On the other side, the LEB band of the 352 nm-CL morphology (Figure 2a) exhibits the longest lifetime. It should also be mentioned that only Figure 2a,f follows an exponential function (straight line in a logarithmic scale). In particular, it is observed that for the HEB, the two polycrystalline layers (550 nm-CS and 352 nm-CL) (see Figure 2b,d) move further and further away from an exponential behavior (i.e., from a straight line in a logarithmic scale) by decreasing the temperature. This might be related to the possible distortions or limitations of the movement of the anions or cations that constitute our perovskite, and it should be understood more deeply.

**Figure 4:**
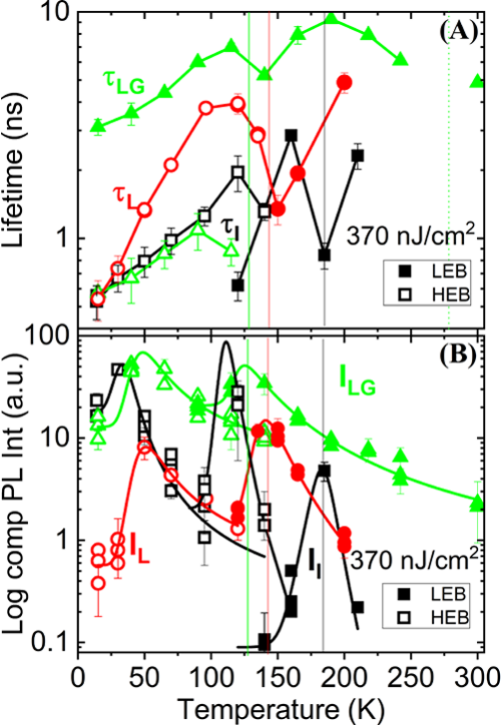
(a) Lifetime as a function of temperature for 352 nm-CL (τ_LG_), 550 nm-CS (τ_L_), and 103 nm-IS (τ_I_). Closed symbols correspond to the data of the LEB and the open symbols to the HEB. (b) Time-integrated PL intensity as a function of temperature for 352 nm-CL (I_LG_), 550 nm-CS and (I_L_), and 103 nm-IS (I_I_). Closed symbols correspond to the data of the LEB and the open symbols to the HEB.

In order to explain the differences of the temporal evolution of each PL contribution/band, we have deduced the “continuous distribution” of characteristic times or lifetimes (see Figure 3 and Figure 4a) that fits the experimental decay curve (Figure 2) by using the Generalized Berberan-Santos decay equation^35^ (see Section 3.2). This characteristic time is an average time of the entire transient extracted from the Berberan-Santos model. Therefore, there is a unique scaling parameter for each PL transient. In the second step, the thermal excitation and the thermal escape were also calculated from the PL intensity plotted in Figure 4b (see Section 3.3). Finally, two fundamental parameters were obtained from the distributions of recombination rate constants: (i) the fast recombination center (*k*_FAST_) and (ii) its dispersion (FWHM). These parameters have provided simple and relevant information to compare the TRPL data for these three morphologies (see Section 3.4).

### 3.2 Continuous Distribution of Characteristic Times: Generalized Berberan-Santos Decay

Assigning an arbitrary number (more than two) of exponential functions to explain the different physical mechanisms involved in carrier recombination can be a speculation. 2-τ exponential fitting can stem from coupled rate equations, but a multi-exponential decay fitting with 3-τ or more or even a non-exponential decay is already very complicated to be supported by an appropriate physical model. Although the use of an arbitrary number of exponential functions is common, it has more physical sense to set the mechanisms and translate them into the appropriate rate equations, whose solution should describe the experimental decay curve. Hence, we have find the “continuous lifetime (τ) distribution” that fits the experimental decay curves by using the Generalized Berberan-Santos equation (GBSe):^30^
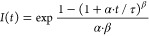
where the scaling parameter τ has dimensions of time. This characteristic time or lifetime is an average time of the entire transient extracted from the GBSe. The shape-determining parameters α and β are dimensionless, with 1 ≥ α > 0 and 1 ≥ β ≥ 0 (see Table 1). If β = 0, a compressed hyperbola function is recuperated, whereas a stretched exponential function is obtained for large values of α and for long times if β > 0. Finally, the Berberan-Santos model behaves as an exponential function for small values of α and/or for values of β near unity. Therefore, this Berberan-Santos relaxation function merges: (i) the compressed hyperbola (or Becquerel^36^) function, which has been used for the description of decays of experimental systems with inorganic solids, and (ii) the stretched exponential (or Kolrausch^37^) function, used for systems with organic molecules.

**Table 1:** Values of the Shape-Determining Parameters α and β and the Corresponding Fitting Types (Fit. T.: Compressed Hyperbola Function (C-Hyp), Stretched Exponential Function (S-Exp), and Exponential Function (Exp)) of the HEB and LEB of the Three Samples

	LG (352 nm-CL)				layer (550 nm-CS)				islands (103 nm-IS)			
EB	*T* (K)	α	β	Fit. T.	*T* (K)	α	β	Fit. T.	*T* (K)	α	β	Fit. T.
HEB	15	0.14(2)	0.0(9)	C-hyp	15	0.000(4)	1.0(2)	Exp	14	0.13(2)	0.00(14)	C-hyp
HEB	40	0.197(10)	0.00(3)	C-hyp	30	0.000(2)	1.0(4)	Exp	30	0.014(5)	1.0(8)	Exp
HEB	65	0.271(7)	0.000(15)	C-hyp	50	0.07(9)	0.00(9)	C-hyp	50	11 × 10^–4^(2)	1.0(9)	Exp
HEB	90	0.316(6)	0.000(3)	C-hyp	70	0.17(2)	0.0(2)	C-hyp	70	2 × 10^–3^(3)	1.0(9)	Exp
HEB	115	0.389(6)	0.000(5)	C-hyp	90	0.02(2)	0.00(2)	C-hyp	95	0.10(2)	1.00(2)	Exp
HEB					120	0.003(5)	0.00(3)	C-hyp	120	0.026(17)	0.0000(9)	C-hyp
HEB									140	0.3(5)	0.00(11)	C-hyp
												
LEB	90	5 × 10^–12^(12)	0.0087(2)	Exp	120	0.04(3)	0.09(6)	S-Exp	120	0.93(8)	0.3(3)	S-Exp
LEB	115	7 × 10^–5^(4)	0.9997(5)	Exp	135	1.0(6)	0.97(8)	S-Exp	140	0.8(4)	0.45(6)	S-Exp
LEB	140	0.05262(2)	1.0(5)	Exp	150	0.02(2)	0.9(2)	Exp	160	1.0(8)	0.69(9)	S-Exp
LEB	165	2 × 10^–4^(3)	0.998(15)	Exp	165	0.04(5)	1.0(2)	Exp	185	0.78(2)	1.00(3)	Exp
LEB	190	0.05(4)	1.00(18)	Exp	200	0.000(7)	1.0(4)	Exp	210	0.31(13)	1.0(2)	Exp
LEB	218	0.1680(5)	1.00(3)	Exp								
LEB	240	0.14922(6)	1.0(3)	Exp								

Our perovskite has both an inorganic framework ({PbI_3_}^−^) and an organic cations MA^+^.

From Table 1, it follows that, for the HEB, i.e., in the orthorhombic phase, the polycrystalline layers (550 nm-CS and 352 nm-CL) behave as a compressed hyperbola (40–120 K). This is consistent with the fact that, for the HEB, the MA^+^ cation becomes fixed, whereas the {PbI_3_}^−^ anion becomes distorted by decreasing the temperature.^7,38−40^ However, the PL transients of the island (103 nm-IS) configuration follow an exponential function (30–95 K), which could be expected in a good quality single crystal of MAPbI3. It could be attributed to the strain relaxation for this morphology and the dominantly free exciton recombination.^1^

On the other hand, the stretched exponential is the predominantly fitting type obtained for the LEB for the two configurations, in which small grains are involved (layer (120–140 K) and islands (120–160 K)). At typical LEB temperatures, the MA^+^ cation shows free rotation, and there exist eight disordered MA^+^ states, whereas the {PbI_3_}^−^ anion becomes less distorted by increasing the temperature.^4,38−40^ Hence, the organic molecules are responsible for this behavior and the phase transitions are linked to the ordered-disordered state of the MA^+^ cation.^41^ However, the PL transients of the configuration of large grains (352 nm-CL) follow an exponential function, which could be due to the large size of the grains. A possible explanation could be, as reported previously, the fact that increasing the grain size from 80 to 352 nm could cause a low density of subgap states at the phase boundaries.^42−44^ This would allow the MA^+^ cations to show free rotation even at low temperatures for larger grains (Figure 2a). However, for the smaller grain size (Figure 2c,e), the temperature should be increased for the PL transient to show an exponential behavior. Therefore, it is expected to obtain the orthorhombic–tetragonal phase transition for the larger grains at a lower temperature rather than for a smaller grain size, as we have found (see Figure 4b). The importance of the grain size has already been demonstrated, for example, to enhance the tetragonal band versus the orthorhombic band^4,43^ or even to produce duplication of bands in other kinds of structures when the periodicity is of the order of 250 nm.^45−47^

The variation of the lifetime against temperature obtained by using the Generalized Berberan-Santos equation is shown in Figure 4a. These values are lower than those reported in the μs region^48−50^ and on the order of 2 ns, which is typically attributed to the direct bandgap semiconductors. It can be recognized as a peculiarity lying in a sharp reversal of the continuous/monotonous variation of the lifetime. These characteristic minima agree with the observed shifts, drops, broadenings, and disappearances of the temperature-dependent data of the PL peak energy of the two bands^4^ and could be originated from the phase transition.^44^ The different lifetimes obtained for the different morphologies by using the GBSe^30^ can be linked to different recombination channels (see Figures 3 and 4a):

1. The emission with the longest lifetime, τ_LG_, on the order of several to 10 ns is dominant in the configuration of large grains (352 nm-CL, see Figure 3 and solid green triangles in Figure 4a). This is due to the fact that a large grain size has a smaller amount of grain boundaries where nonradiative recombination can take place.^51,52^ As a result, a long lifetime is induced by a reduction of nonradiative recombination channels, which is due to a reduced number of trap states at the grain boundaries.^35,53,54^
2. The lifetime τ_L_ (in between 1.3 and 5 ns) is related to recombination channels associated to defective-“T” states originated by possible strain inhomogeneities (see Figure 3 and solid red circles in Figure 4a).^1^
3. A fast lifetime τ_I_ = 0.5–1.3 ns was observed only at the low-density dispersive isolated-island configuration 103 nm-IS (see Figures 3 and 4a). In this case, the strain relaxation isolated-islands have no traces of shallow electronic levels due to defective-“T” states.^1^ We can refer the reader here to a previous work for a better understanding of the role of strain relaxation, “trap states”, etc. in these samples.^1^ Additionally, as observed in Figure 4a, the shortest photoluminescence lifetime was obtained for individual isolated MAPbI_3_ islands as a result of great separation between the islands.

To sum up, the lifetime differences are due to changes in a nonradiative mechanism relative to the boundaries of the grain, whose surface is the one that defines its contribution with respect to radiative recombination. Hence, a long lifetime, τ_LG_, is obtained in the configuration of large grains (352 nm-CL) due to a reduction of the nonradiative recombination as a result of a reduced number of trap states at the grain boundaries. Moreover, the lifetime is also due to the levels of carrier capture, which are responsible for the increase in time with temperature. Hence, even though the layer (550 nm-CS) and the island (103 nm-IS) films have the same small grain size (103 ± 9 nm) and therefore the same boundaries of the grain, only the layer (550 nm-CS) configuration has defective-“T” states originated by strain inhomogeneities.

Thus, the observed differences in the photoluminescence lifetimes (ranging from 0.5 ns to few nanoseconds for 370 nJ/cm^2^) are due to the boundaries of the grain and the levels of carrier capture. Hence, the island (103 nm-IS) configuration has smaller lifetimes due to the small grain size (or large boundaries of the grain) and its lack of connectivity (avoiding diffusion) that causes the strain relaxation. A general picture of the mechanisms explaining carrier recombination dynamics in MAPbI_3_ can be found in Figure 2 of ref (55).

### 3.3 Thermal Excitation and Thermal Escape

In Figure 4b, we examine the phase transition regime more closely by plotting the time-integrated PL intensity of a normalized decay against temperature for the two energy bands. By increasing the temperature for an energy band, at least one peak of the time-integrated PL intensity is obtained. This fact has been linked to a phase transition.

For the high energy band (orthorhombic phase), a low temperature phase transition/discontinuity is observed at around 50 K (for 550 nm-CS and 352 nm-CL).^1,4^ The increase in intensity from 15 to 50 K of almost one order of magnitude should be noted. For 103 nm-IS, two (30 and 110 K) phase transitions/discontinuities below the expected orthorhombic–tetragonal phase transition (see black, red, and green lines in Figure 4b) were observed. Note that the HEB is observed from 15 to 140 K for this configuration. Although there are some experimental data, a great difference in intensity is again appreciated, especially for the second maximum between the data at 95 K and the one at 120 K.

For the low energy band (tetragonal phase), we have obtained the characteristic orthorhombic–tetragonal phase transitions at 145 K for 550 nm-CS and around 127 K for 352 nm-CL (see Section 3.2), but also, another phase transition/discontinuity at a higher temperature (180 K for 103 nm-IS) than the typical orthorhombic–tetragonal phase transition (127–140 K) has been obtained for the polycrystalline layers (550 nm-CS and 352 nm-CL).

The three different morphologies show maximum emission centered at different (transition) temperatures for the same laser excitation, where this temperature is mainly determined by the speed of the process.^56−63^ Interestingly, the configuration that exhibits shorter lifetimes than the other configurations for the PL intensity, as it is the case of the 103 nm-IS, is the first one to undergo the phase transitions (by decreasing the temperature). This can be understood if we consider that the phase transformation will be more homogeneous in the case of 103 nm-IS because of the strain relaxation isolated islands. Thus, from a broader perspective, our study shows that the less extensive the limits of grain and subgrains and the less connected the morphologies, the faster the process of phase transformation.^1,4,64^ Hence, only in the case of 103 nm-IS that no crystallographic phases coexist,^1^ which has been observed in the other two configurations (550 nm-CS and 352 nm-CL)^1,4^ as well as in several inorganic perovskite materials.^65−70^ Despite the fact that in our two polycrystalline layers (550 nm-CS and 352 nm-CL), the HEB and the LEB seem to coexist in an intermediate temperature range, the decrease in the HEB could be at the expense of the increase in the LEB, if we assume that both phases have thermal connection, and it could be considered as a non-radiative decrease instead of as the peak of the phase transition (50 K).

The representation of the log component PL intensity versus temperature (Figure 4b) allows us to calculate both the activation energies for the thermal escape (*E*_act_) and thermal excitation (*E*_low_) for each of the two energy bands since the PL intensity can be described as follows:^48,49^
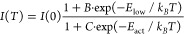
where *B* and *C* are the proportionality factors. The estimated *E*_low_ and *E*_act_ values of the HEB and LEB are presented in Table 2.

**Table 2:** Estimated *E*_low_ and *E*_act_ Values of the HEB and LEB of the Three Samples

	1st phase trans. 20–60 K		2nd phase trans. 96–150 K	
	*E*_low_ (meV)	*E*_act_ (meV)	*E*_low_ (meV)	*E*_act_ (meV)
ISLANDS-SG	13(4)	32(8)	292(40)	494(50)
LAYERS-SG	26(13)	43(11)	287(60)	420(80)
1LAYER-LG	25(5)	40(9)	280(80)	340(90)

The first phase transition/discontinuity (20–60 K) has energy values of the order of the tens of meV and the second phase transition (96–150 K) of the hundreds. The thermal escape energy (*E*_act_) is less than three times larger than the thermal excitation energy (*E*_low_). The isolated-island configuration, in which PL transients follow an exponential function instead of a compressed hyperbola function, has lower values at the first phase transition, possibly consistent with the small lifetimes. Its behavior at low temperatures would be that expected in a good quality single crystal of MAPbI_3_.^71^

The estimated *E*_low_ and *E*_act_ of the LEB have lower values at the orthorhombic–tetragonal phase transition for the configuration of large grains (352 nm-CL), in which the PL spectra follow an exponential function instead of a stretched exponential function, but at that time, it exhibits long lifetimes. Hence, this promising result of our work clearly shows that the isolated-island (103 nm-IS) configuration is the first to undergo the O–T phase transition by decreasing the temperature, with shorter lifetimes; meanwhile, the large grain configuration (352 nm-CL) undergoes the O–T phase transition at a lower temperature, with longer lifetimes. Thus, this is consistent with the fact that the less extensive the limits of grain and subgrains and the less connected morphologies, the faster the process of phase transformation. These results give further insights into the photoluminescence response to the crystallite dimensions, connectivity, and perovskite crystal phases.

### 3.4 Distribution of Recombination Rate Constants

**Figure 5:**
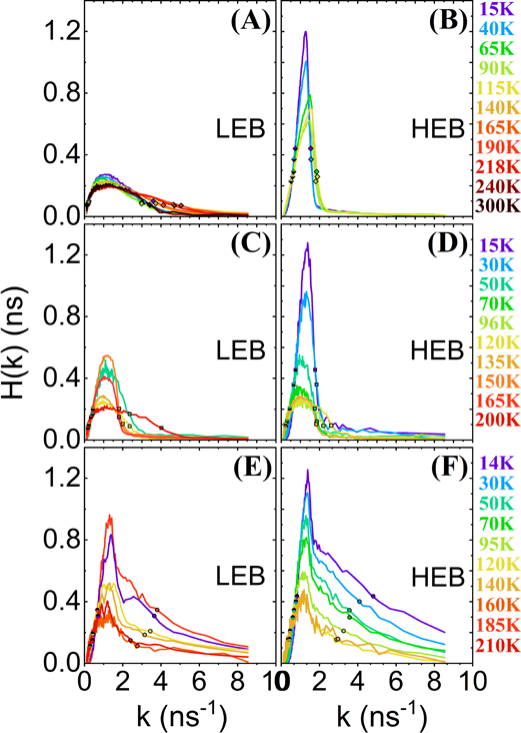
(a–f) Distributions of the recombination rate constants of the three different morphologies: (a, b) one layer of large grains (352 nm-CL), (c, d) multilayers of small grains (550 nm-CS), and (e, f) one layer of islands of small grains (103 nm-IS). The areas of the distribution of recombination centers are normalized to 1. The diamonds, squares, and circles represent the slow and fast recombination centers of 352 nm-CL, 550 nm-CS, and 103 nm-IS, respectively.

The experimental time-resolved photoluminescence emission curve *I*(*t*), plotted in Figure 2, has been normalized such that the area under it is equal to 1. By directly inverting (*k* = 1/τ) the *I*(*t*)/area, we can obtain the normalized area probability density function *H* (ns) as a function of the distribution of recombination rate constants (*k* (ns^–1^)), as shown in Figure 5.^35^ Two fundamental parameters can be obtained: (i) its dispersion (FWHM), as if *H*(*k*) was a single band, without considering the narrow contribution of the islands (103 nm-IS) configuration and (ii) the fast/slow recombination center (*k*_FAST_ and *k*_SLOW_), defined as:
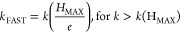

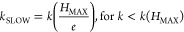


**Figure 6:**
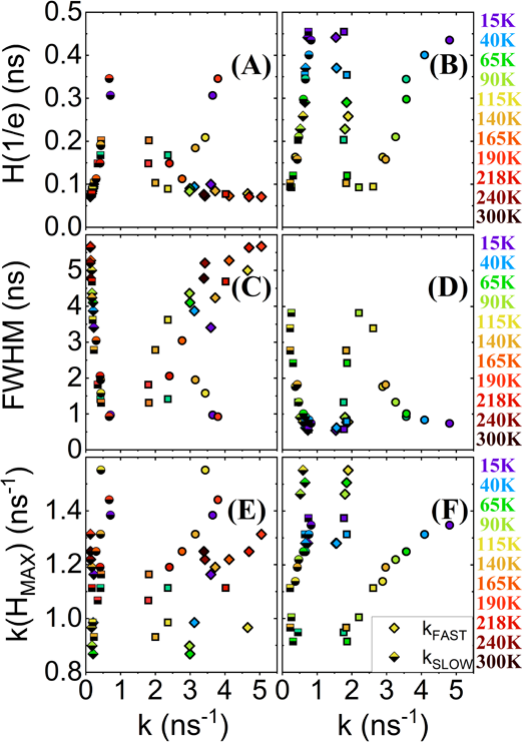
Comparison of slow and fast recombination centers (*k*_SLOW_ and *k*_FAST_) in absolute number for the three configurations as a function of (a, b) *H*(1/*e*), (c, d) FWHM, and (e, f) *k*(*H*_MAX_). The diamonds, squares, and circles represent the slow and fast recombination centers of 352 nm-CL, 550 nm-CS, and 103 nm-IS, respectively.

These definitions provide simple and relevant information to compare the three perovskite morphologies, as well as for LEB and HEB PL contributions (see Figure 5a–f). Considering that the areas of the distribution of recombination centers are normalized to 1, it can be deduced from Figure 5 that the three samples are not only distinguished due to different (small/bigger) grain size ratios since the maximum of the probability density function (*H*_MAX_) does not match that at the same *k*(*H*_MAX_) for a given temperature. Additionally, *H*_MAX_ is smaller for the LEB than for the HEB, especially for large grains, 352 nm-CL, where the *H*_MAX_ is significantly smaller for the LEB (<0.3 ns for all temperatures) and the fast recombination centers (*k*_FAST_) are larger (Figure 5a,b). If we take a closer look at these parameters (see Figure 6a–f), we observe that the three samples are almost identical in their behavior at long lifetimes (small *k*, *k*_SLOW_). In addition, at low temperatures, they present smaller *k*_SLOW_. However, the 550 nm-CS sample (at HEB) and the 352 nm-CL sample (at LEB) have fewer slow recombination centers in absolute number compared to the other two samples, with 103 nm-IS being the sample that presents more slow recombination centers (*k*_SLOW_).

The behavior at short lifetimes (large *k*, *k*_FAST_) is clearly different for the three samples. A large number of fast recombination centers for the LEB and for the HEB are obtained in the configuration in which *E*_low_ and *E*_act_ are lower: (i) At the LEB, the largest number of fast recombination centers (*k*_FAST_) has been observed for 352 nm-CL (Figure 6a,e) and (ii) at the HEB, a very large number of widely dispersed fast recombination centers (*k*_FAST_) for sample 103 nm-IS in comparison to the other two polycrystalline samples have been observed (Figures 6b,f).

These two configurations (103 nm-IS and 352 nm-CL) are also the only ones that follow an exponential function, as seen in Section 3.2.

To sum up, for the isolated-island configuration at the HEB, (i) the PL transients follow an exponential function instead of a compressed hyperbola function, (ii) it has the lowest thermal excitation (*E*_low_) and the thermal escape (*E*_act_) values at the first phase transition, (iii) it has the smallest lifetime, (iv) it has the largest *H*(1/*e*), and (v) the fast recombination rate centers are up to 4.8 ns^–1^, compared to less than 2 ns^–1^ for the other two configurations. This represents a clear break in the behavior of the isolated-island configuration (see Figure 5f). All these results are consistent with each other and stem from the strain relaxation that prevents shallow electronic levels from arising due to defective-“T” states.

## 4 Conclusions

PL measurements for the three morphologies in polycrystalline thin film configuration at varying temperatures (14–300 K) evidenced characteristic differences on the electronic properties of lead iodide perovskite. In this work, we applied the Generalized Berberan-Santos model to analyze the PL decay to account the short photoluminescence lifetimes (nanosecond) since the models of quantum confinement or surface chemistry cannot completely explain our results. We found that the PL transient of the high energy band (HEB) in the orthorhombic phase of polycrystalline layer morphology decayed as a compressed hyperbola (40–120 K), consistent with the fact that the MA^+^ cation becomes fixed, whereas the {PbI_3_}^−^ octahedral anion becomes distorted by decreasing the temperature. Instead, PL transients of the island configuration follow an exponential function decay attributed to strain relaxation and dominantly free exciton recombination as expected in a good quality single crystal of MAPbI_3_. On the other hand, the low energy band (LEB) decayed following the stretched exponential function only if the sample contained small crystallites. The island morphology-type film implies larger boundaries of the grain with an increased number of trap states on boundaries compared to larger crystallite morphologies. Then, faster recombination rate centers are numerous and widely dispersed producing short photoluminescence lifetimes, low thermal excitation, and thermal scape due to this characteristic broken connectivity between grains.
